# A novel voice in head actor critic reinforcement learning with human feedback framework for enhanced robot navigation

**DOI:** 10.1038/s41598-025-92252-w

**Published:** 2025-02-28

**Authors:** Alabhya Sharma, Ananthakrishnan Balasundaram, Ayesha Shaik, Chockalingam Aravind Vaithilingam

**Affiliations:** 1 School of Computer Science and Engineering, Vellore Institute of Technology, Chennai, 600127 India; 2 Centre for Cyber Physical Systems, Vellore Institute of Technology, Chennai, 600127 India; 3 Clean Technology Impact Lab, Taylor’s University, 47500 Subang Jaya, Malaysia

**Keywords:** Navigation and mapping, Large Language models, Reinforcement learning, Artificial general intelligence, Robotics, Computer science, Engineering

## Abstract

This work presents a novel Voice in Head (ViH) framework, that integrates Large Language Models (LLMs) and the power of semantic understanding to enhance robotic navigation and interaction within complex environments. Our system strategically combines GPT and Gemini powered LLMs as Actor and Critic components within a reinforcement learning (RL) loop for continuous learning and adaptation. ViH employs a sophisticated semantic search mechanism powered by Azure AI Search, allowing users to interact with the system through natural language queries. To ensure safety and address potential LLM limitations, the system incorporates a Reinforcement Learning with Human Feedback (RLHF) component, triggered only when necessary. This hybrid approach delivers impressive results, achieving success rates of up to 94.54%, surpassing established benchmarks. Most importantly, the ViH framework offers a modular and scalable architecture. By simply modifying the environment, the system demonstrates the potential to adapt to diverse application domains. This research provides a significant advancement in the field of cognitive robotics, paving the way for intelligent autonomous systems capable of sophisticated reasoning and decision-making in real-world scenarios bringing us one step closer to achieving Artificial General Intelligence.

## Introduction

Advancements in cognitive robotics are reshaping the perception capabilities of intelligent systems, heralding a new era in autonomous system design yet the quest to endow robots with human-like cognition and perception remains an intimidating challenge^1^. Despite these significant advancements, replicating the intricate mechanisms of human perception and decision-making in robots poses profound theoretical and practical challenges due to their data driven nature. The primary focus driving this research to integrate and advance current technologies to enhance cognitive and perceptive capabilities in autonomous systems, moving closer to achieving Artificial General Intelligence (AGI).

The challenge in cognitive robotics centres on developing systems capable of advanced perception and interaction within their environments. The field has evolved from basic task-oriented robots to complex systems requiring sophisticated sensory processing and decision-making capabilities. Key research areas in this domain include robotic mapping, perception, and interaction, with a focus on integrating semantics to enhance these functions^2^. The integration of AI and robotics has been extensively studied in^3,4^ providing foundational insights into the potential of AGI in robotics. Additionally, the rapid progress in multimedia acquisition technology and the introduction^5^ of AI theory have made visual perception a hot topic in both academic and industrial applications, especially in areas like product surface defect detection and intelligent agricultural production^6^.

This work addresses current limitations in robotic systems to achieve human-like cognition and perception. The integration of learning systems, such as deep learning and reinforcement learning, has significantly improved visual-based self-state estimation, environment perception, and navigation capabilities in autonomous systems (Tang et al., 2022). However, bridging the gap between current technological capabilities and the ambitious goal of AGI remains a key challenge. Recent overviews of AGI have highlighted the progress and future challenges in this area, reflecting the ongoing efforts to develop intelligent systems with generalizable capabilities akin to human intelligence^7^.

Generative AI models like ChatGPT^8^ have catalysed development in AI, showcasing the potential of AI in application development and problem-solving, thus contributing to the evolution of cognitive robotics^9^. The exploration of AGI encompasses the development of systems that can perform a wide range of tasks, adapt to new environments, and learn from experiences in a manner like humans. This involves not only advancements in AI and machine learning but also breakthroughs in understanding and replicating human cognition and perception.

The research presented in this work aims to synthesize the current trends and methodologies in cognitive robotics, focusing on how the integration of advanced AI systems, machine learning models, and perception technologies can enhance cognitive capabilities in robots. By analysing the recent developments and future challenges in AGI, the work seeks to provide insights into the potential pathways towards realizing more advanced and contextually aware autonomous systems. This introduction sets the stage for a comprehensive exploration of the advancements in semantic SLAM, LLMs and RL in the field of robotics^10,11^, as detailed in the subsequent sections of this manuscript. Specifically, the research has the following contributions:

- This work proposes a novel actor-critic RL model incorporating two LLMs for enhanced perception.
- A point cloud-based mapping system with appropriate triggers for various use cases while handling edge cases has been developed.
- The proposed actor-critic RL model is integrated with a Proximal Policy Optimization (PPO) algorithm which will control the environment mapping.
- A seamless query-response pipeline powered by an Object-oriented methodology with semantic search capabilities has been developed.

The manuscript is further organized such that Section “Related work” discusses the contemporary works followed by Section “Environment setup” discussing the environmental setup for performing this research work. Section “Proposed system architecture” discusses the proposed work, Section “Experimental results” provides the experimental results followed by Section “Discussion” discussing in detail about the inferences from the results. Finally, the conclusion is provided in Section “Conclusion”.

## Related work

The integration of semantic SLAM, LLMs and RL is revolutionizing the perception capabilities of intelligent systems, particularly in robotics. This literature review focuses on how these technologies collectively contribute to the development of advanced robotic systems. Subsequent sections of this literature review detail the advancements and interplay of semantic SLAM, LLMs, and RL in the field of robotics. Starting from foundational developments in natural language processing with InstructGPT by^12^ a fine-tuned version of GPT-3, which marks a significant advancement in aligning AI with human preferences in NLP tasks^13^. The model’s improved ROUGE-L and Perspective API scores over the 175B GPT-3 model reflect its enhanced accuracy and reliability. This foundation is further strengthened by^14^, who present an environment representation model integrating perception and semantics. The model’s unique approach to ontology construction from WordNet glosses is a testament to its efficacy. However, the generalizability of these models across diverse real-world scenarios remains a gap in research^15^. extend this by exploring semantic perception in humanoid robots, achieving an impressive 85% accuracy in recognizing and executing human activities^16^. contribute to this theme with LM-Nav, a system adept at navigating complex environments using pre-trained models, highlighting the practical applicability of NLP in robotics.

Transitioning to a more complex domain^17^ introduce LP-SLAM, a novel SLAM system that leverages LLMs for text landmark detection. Achieving an average ATE of 0.05 m on the TUM RGB-D dataset, LP-SLAM exemplifies the integration of NLP and SLAM. This integration is further seen in the work of^18^ who proposed object-oriented semantic mapping, achieving an average precision of 0.84 in object detection on the NYUv2 dataset. However, the application of these technologies in dynamic, real-world environments introduce new challenges, such as maintaining accuracy and computational efficiency. Nielsen et al., 2023 feature-based SLAM in non-static environments and^19^ perception-aware planning for active SLAM with MAVs represent significant advancements in addressing the dynamic nature of real-world settings^20,21^. Further this exploration by enhancing the adaptability of SLAM systems to environmental changes and integrating visual perception with contextual semantics.

The review culminates in an examination of sophisticated applications, where Nvidia (Ma et al., 2023) integrate LLMs with RL Eureka, demonstrating its superior performance in 83% of the tasks across 29 RL environments. This integration highlights the potential of AI systems in learning complex skills autonomously. HELM by^22^ introduces history compression via language models in Monte Carlo based RL, showing 10 times increase in sample efficiency^23,24^. further advance the SLAM field, with Dynamic-SLAM enhancing localization and mapping accuracy in mobile robots, and the integration of semantics and visual information opening new frontiers in autonomous systems design. Similarly^25^, propose a deep learning-based approach integrating LSTM and DDPG to mitigate time delays in telepresence robots, achieving a 2.3% improvement in response time and enhancing control during communication lapses^26^. work on semantic mapping on mobile robots underscores the growing trend of semantic understanding in robotics. The works carried out by^27–29^ discuss about robotic assistance in application areas such as tele-health and rehabilitation using deep RL.

The impressive capabilities of DeepMind’s Gemini^30^, as evidenced by its performance against GPT-4 on MMLU (90% vs. 86.4%), Big-Bench Hard (83.6% vs. 83.1%), HumanEval (74.4% vs. 67%), and VQAv2 (77.8% vs. 77.2%), set a benchmark in AI’s multimodal versatility. Although Gemini does not directly incorporate SLAM, its achievements provide a roadmap for integrating advanced AI systems with SLAM technologies^31^. propose ConceptGraphs, a framework utilizing open-vocabulary 3D scene graphs for robot perception and planning. Their approach leverages semantic information from LLMs to encode scene elements and their relationships. This enables robots to localize within known environments and map new objects using zero-shot detection and segmentation models. Additionally, LLM embeddings allow for text-based queries within the 3D scene, facilitating interaction between robots and the environment. While demonstrating strong performance, ConceptGraphs does not explicitly explore LLM-based planning over the 3D scene graphs. Furthermore, the computational demands of the proposed tech stack may limit its applicability on mobile robots in real-world scenarios. A more LLM oriented planning approach is proposed by^32^ introducing SayPlan, an LLM-based framework for robotic task planning in home environments. SayPlan leverages LLMs for semantic search and plan generation. It inherits biases and inaccuracies common to LLMs and struggles with distance, count, and negation-based reasoning. Additionally, SayPlan assumes a static pre-mapped environment limiting its real-world adaptability.

The aim of this work is to harness methodologies like those in ConeceptGraphs, SayPlan, Eureka and others reviewed herein to build more sophisticated approach for cognitive systems. By combining the strengths of multimodal AI and SLAM, the future of robotic perception and autonomy looks promising, with the potential to develop systems capable of complex interactions and enhanced decision-making in dynamic environments. This integration signifies a pivotal step towards realizing more advanced and contextually aware autonomous systems, bringing us one step closer to Artificial General Intelligence.

## Environment setup

To emulate real-world conditions and facilitate the operation of autonomous virtual robots, we employed advanced physics simulators available in select game engines. These simulators offer high-fidelity 3D environments, crucial for testing and validating our proposed framework in various applications and generalized scenarios. Given the project’s primary implementation in Python, establishing seamless communication between the Python codebase and the virtual robot within the simulation environment while having a control over data that can be accessed by the agents is important.

### Unreal engine 5.3

Unreal Engine (UE) 5.3 emerged as a prime choice for its robust physics simulation capabilities and unparalleled graphical fidelity. Leveraging its cutting-edge features, we were able to create immersive and realistic environments that closely resemble real-world scenarios using Unreal Engine, 2024. Despite not directly controlling the environment, Unreal Engine provided a versatile platform for designing intricate scenes and scenarios for our experiments. We utilized Unreal Engine’s Blueprint system to define complex interactions and events within the environment, allowing for dynamic changes and scenarios during simulation runs.

### Microsoft airsim plugin

**Fig. 1:**
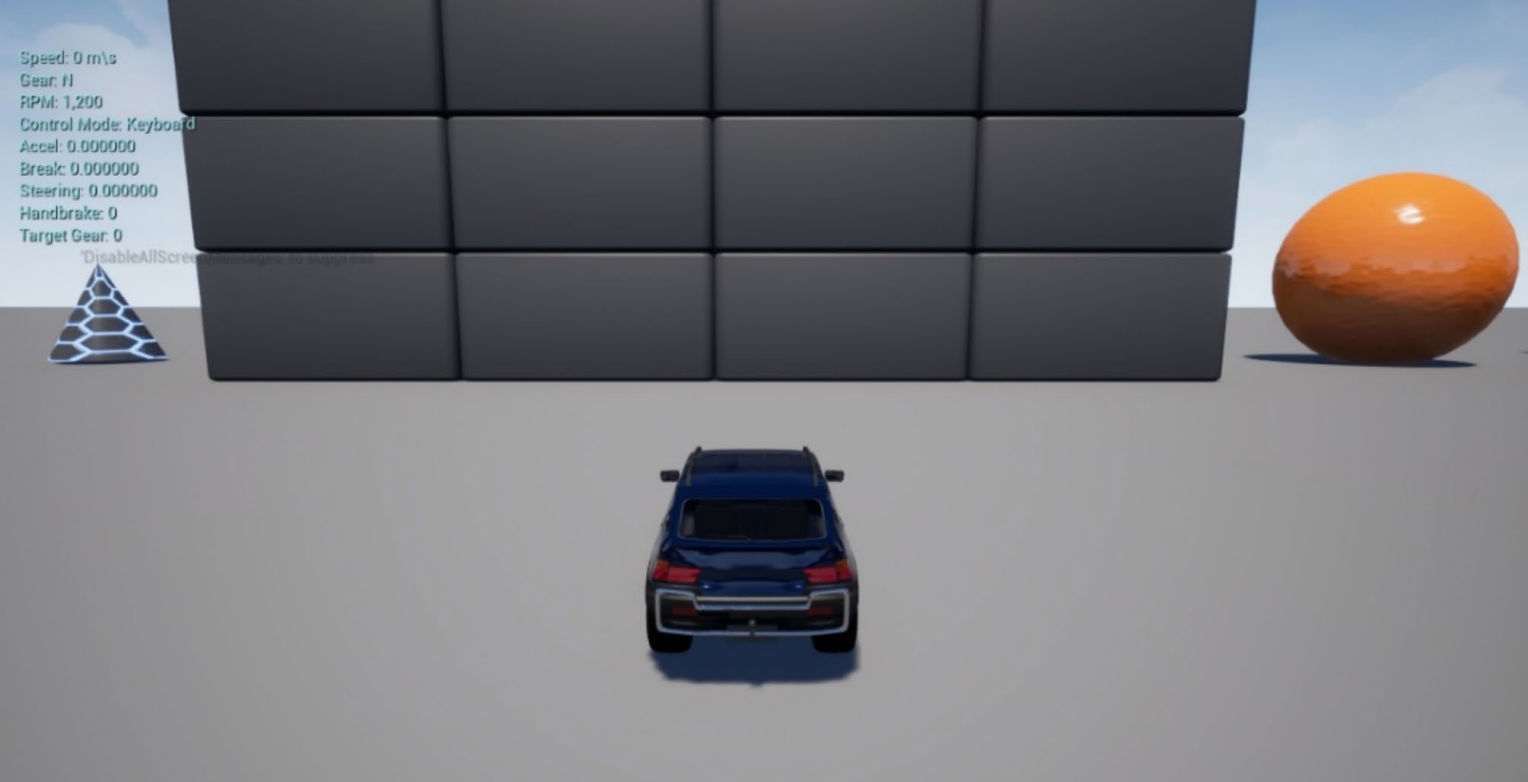
Blocks environment on UE 5 with Vehicle Client^10^.

In conjunction with our environment setup, we integrated the Microsoft AirSim plugin^33^, a sophisticated tool tailored for simulating drones and autonomous vehicles. While the Unreal Engine served as the graphical rendering engine, the AirSim plugin facilitated the nuanced simulation of vehicles and their interactions within the virtual environment as shown in Fig. 1. Offering a standardized interface, the AirSim plugin enabled seamless interaction with simulated vehicles, including drones and ground vehicles, within the Unreal Engine environment. Furthermore, it provided realistic physics and sensor simulations crucial for evaluating perception algorithms.

However, it is pertinent to note that Microsoft has discontinued further development of the AirSim plugin, resulting in its deprecation. Despite this, the open-source community, particularly projects such as Colosseum, 2024, has undertaken the responsibility of maintaining and updating the AirSim plugin. This community-driven initiative ensures its compatibility with newer versions of the Unreal Engine and sustains support for users engaged in simulation-based research and development within the fields of robotics and autonomous systems. Although the environmental attributes remained static, the AirSim plugin facilitated dynamic control and manipulation of virtual agents (robots) within the simulated environment. Leveraging the AirSim APIs, we put together the behaviour and actions of virtual robots, thereby enabling realistic navigation, perception, and interaction tasks within the simulated domain.

### Computational requirements and scalability

Both Unreal Engine 5.3 and the Microsoft AirSim plugin require high computational power for optimal performance. Our simulations were run on a system equipped with an Intel Core i7 processor, 16 GB of RAM, an NVIDIA GeForce RTX 3060 graphics card, and an SSD for efficient data access and storage. This setup provided the necessary computational resources for smooth rendering of high-fidelity graphics, real-time physics simulations, and seamless interaction between the Python codebase and the simulation environment. The scalability of Unreal Engine allows for adjustments in graphical fidelity and simulation complexity, ensuring broad accessibility across various hardware configurations. The AirSim plugin also supports scalability, allowing modifications in vehicle models, sensor configurations, and data fidelity to maintain performance on different systems.

## Proposed system architecture

**Fig. 2:**
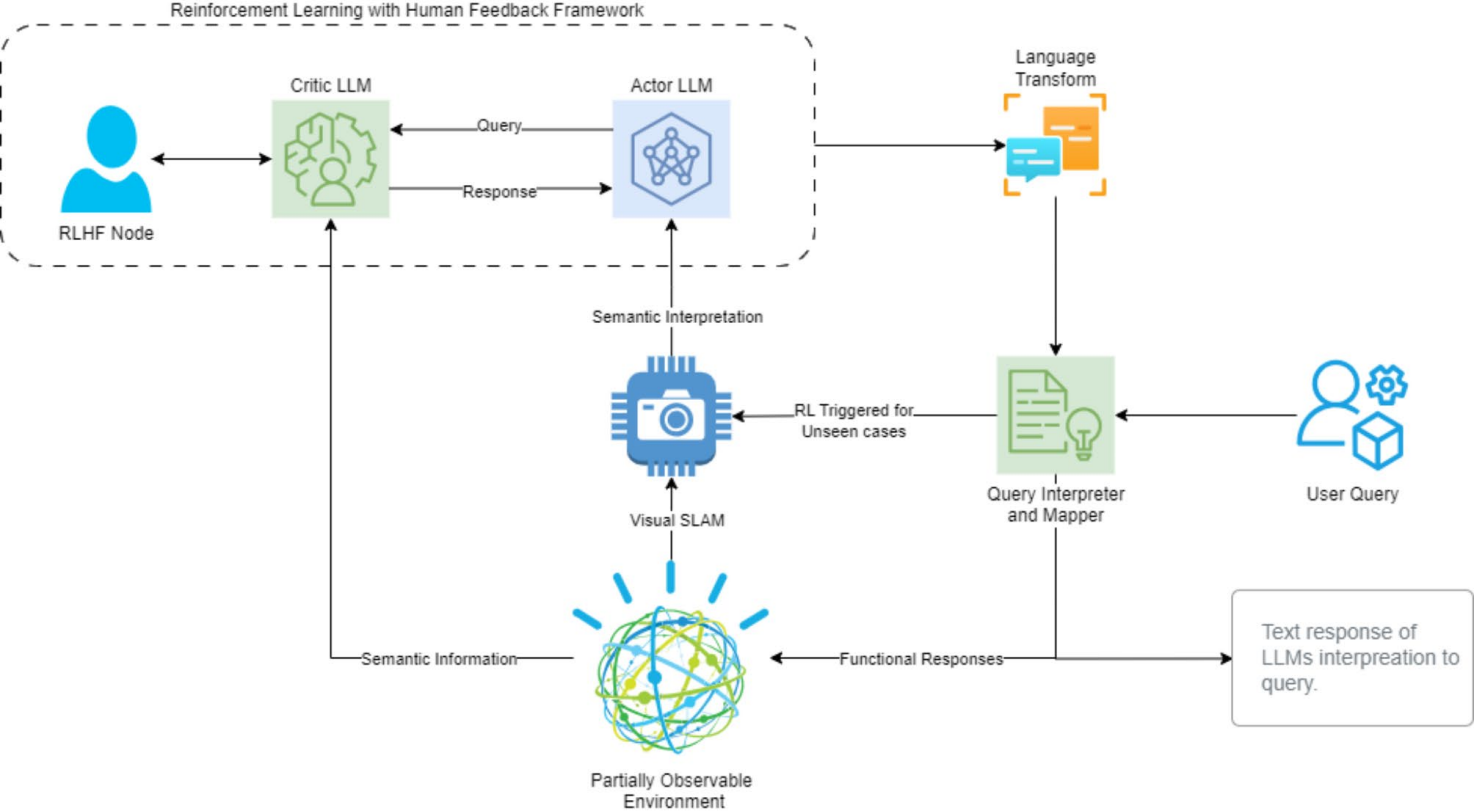
Architecture of the proposed system.

Our proposed architecture aims to elevate the cognitive capabilities of autonomous systems by seamlessly integrating actor-critic RL, powered by LLMs. This system navigates partially observable environments, utilizing object-oriented semantic properties for context-aware decision-making. Our research focuses on leveraging LLMs’ advanced natural language processing (NLP) abilities to enhance existing perception approaches. Building upon AirSim’s Python scripts we intend to demonstrate how LLM-driven agents can optimize an autonomous system’s understanding of its surroundings. we’ve developed a novel system architecture comprised of several interconnected modules, as illustrated in Fig. 2. Each module plays a critical role in enabling the system’s intelligent behaviour. We’ll delve deeper into each module and its function in the following subsections.

### Point clouds mapping

**Fig. 3:**
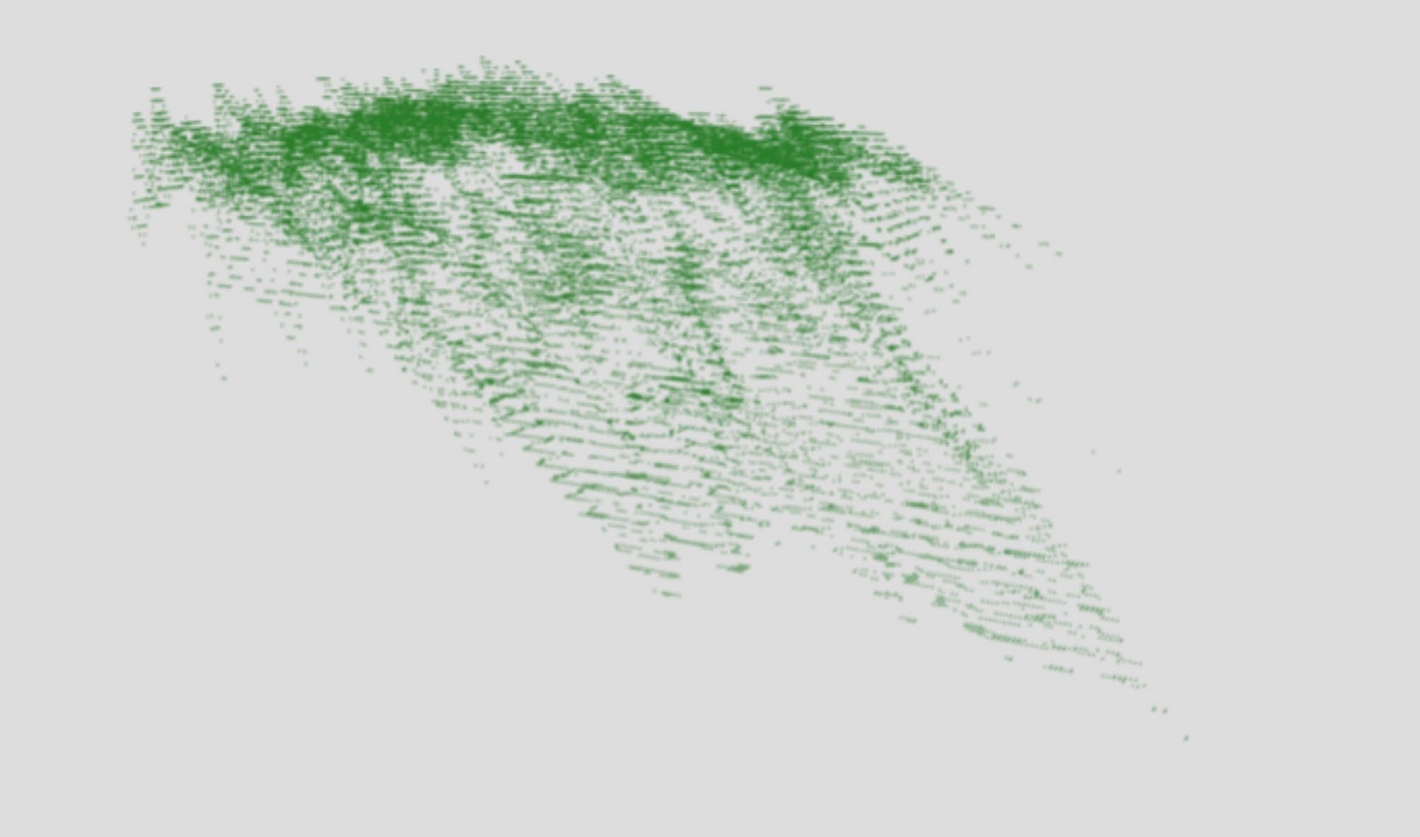
A point cloud of objects in front of the agent using LIDAR data.

Our system’s environmental perception begins with the generation of a 3D point cloud. We capture lidar sensor data, represented as a set of 3D coordinates: *P* = {(*x*_*i*_ ​,*y*_*i*_​, *z*_i_)} for *I* = 1,2,…,*n*, where *n* is the number of points in the point cloud. To model the spatial distribution of these points around the agent’s position, we utilize a multivariate Gaussian distribution. This allows us to estimate *f*_*lidar*_ in Eq. 1, where the amplitude (A) and standard deviations (σ) of the distribution shape the point cloud’s structure. As visualized in Fig. 3, this phase provides the fundamental geometric presentation of the environment, upon which our system’s understanding is progressively refined.

1 $$f_{{lidar}} \left( {x,y,z} \right) = \mathop \int \limits_{{i = 1}}^{n} A_{i} \cdot ~exp^{{\left( { - \frac{1}{2}\left[ {\frac{{\left( {x - x_{i} } \right)}}{{\sigma _{x}^{2} }}~ + ~\frac{{\left( {y - y_{i} } \right)}}{{\sigma _{y}^{2} }}~ + \frac{{\left( {z - z_{i} } \right)}}{{\sigma _{z}^{2} }}} \right]} \right)}}$$

**Fig. 4:**
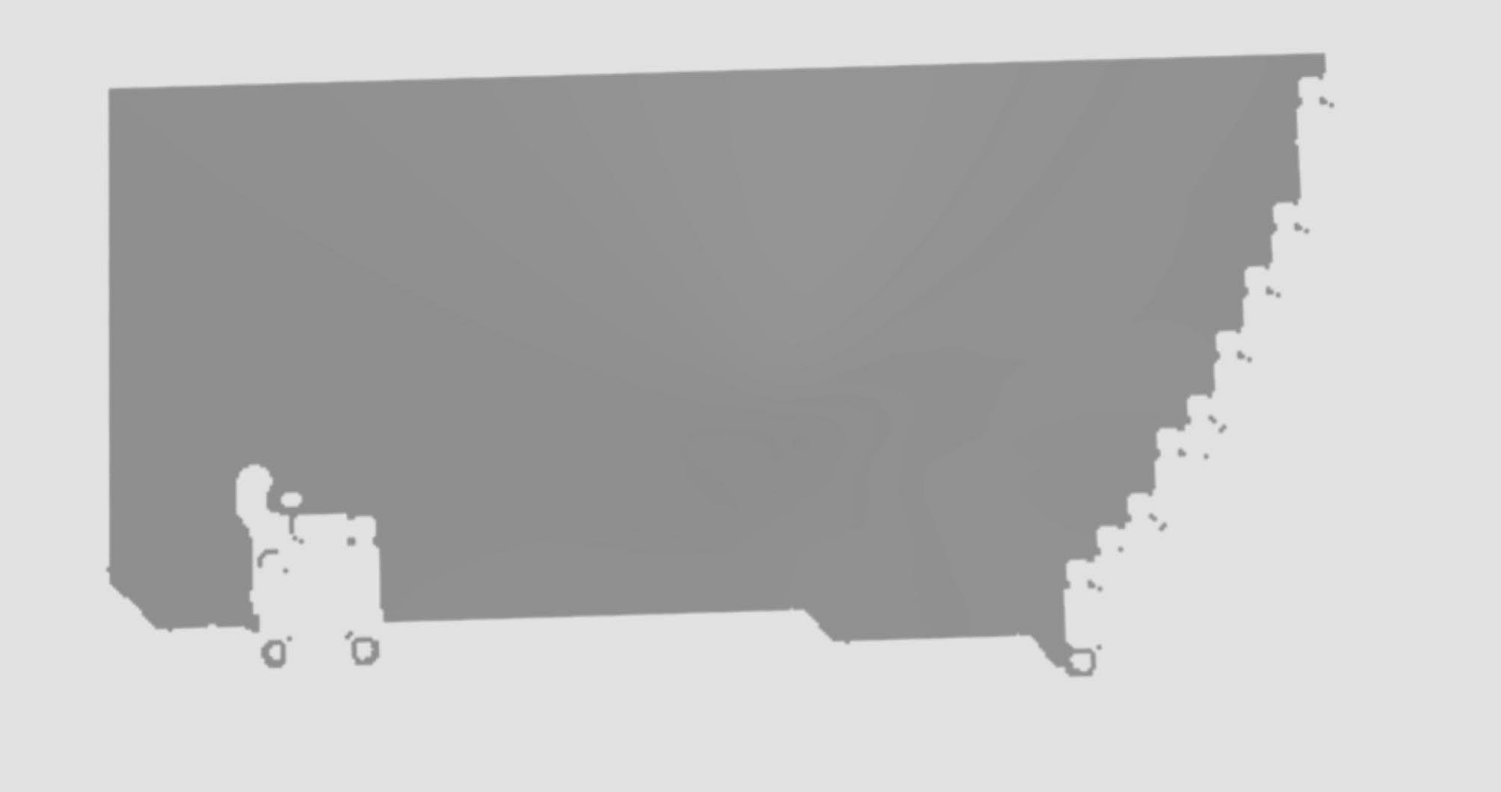
Mapping RGB image using point clouds from LIDAR sensor and depth perception.

We augment the initial point cloud to achieve denser coverage and incorporate visual information. First, the AirSim API’s depth perspective image is decoded and converted it to grayscale. Next, 2D image is reprojected into 3D space using a projection matrix as shown in Eq. 2. Combining it with the initial point cloud, results in a denser representation called *f*_*dense*_. This denser point cloud retains the geometric accuracy of the lidar measurements while incorporating additional detail from the depth image. Finally, we apply a colormap function to assign RGB colours to each point based on its corresponding image pixel using Eq. 3, enhancing the visual quality of the point cloud (as shown in Fig. 4).

2 $$\:{f}_{3D}\left(x,y\right)=\left[\begin{array}{cccc}-0.501202762&\:0.0&\:0.0&\:0.0\\\:0.0&\:\:-0.501202762&\:0.0&\:0.0\\\:0.0&\:0.0&\:10.0&\:100.0\\\:0.0&\:0.0&\:-10.0&\:0.0\end{array}\right]\left[\begin{array}{c}x\\\:y\\\:depth(x,y)\\\:1\end{array}\right]$$

3 $$\:C(png,{x}_{i},{y}_{i})=\left\{\begin{array}{c}\left[{r}_{i},{b}_{i},{g}_{i}\right],\:\:{x}_{i}{,y}_{i}\:valid\:in\:png\\\:[0,\:0,\:0],\:\:otherwise\end{array}\right.$$

**Fig. 5:**
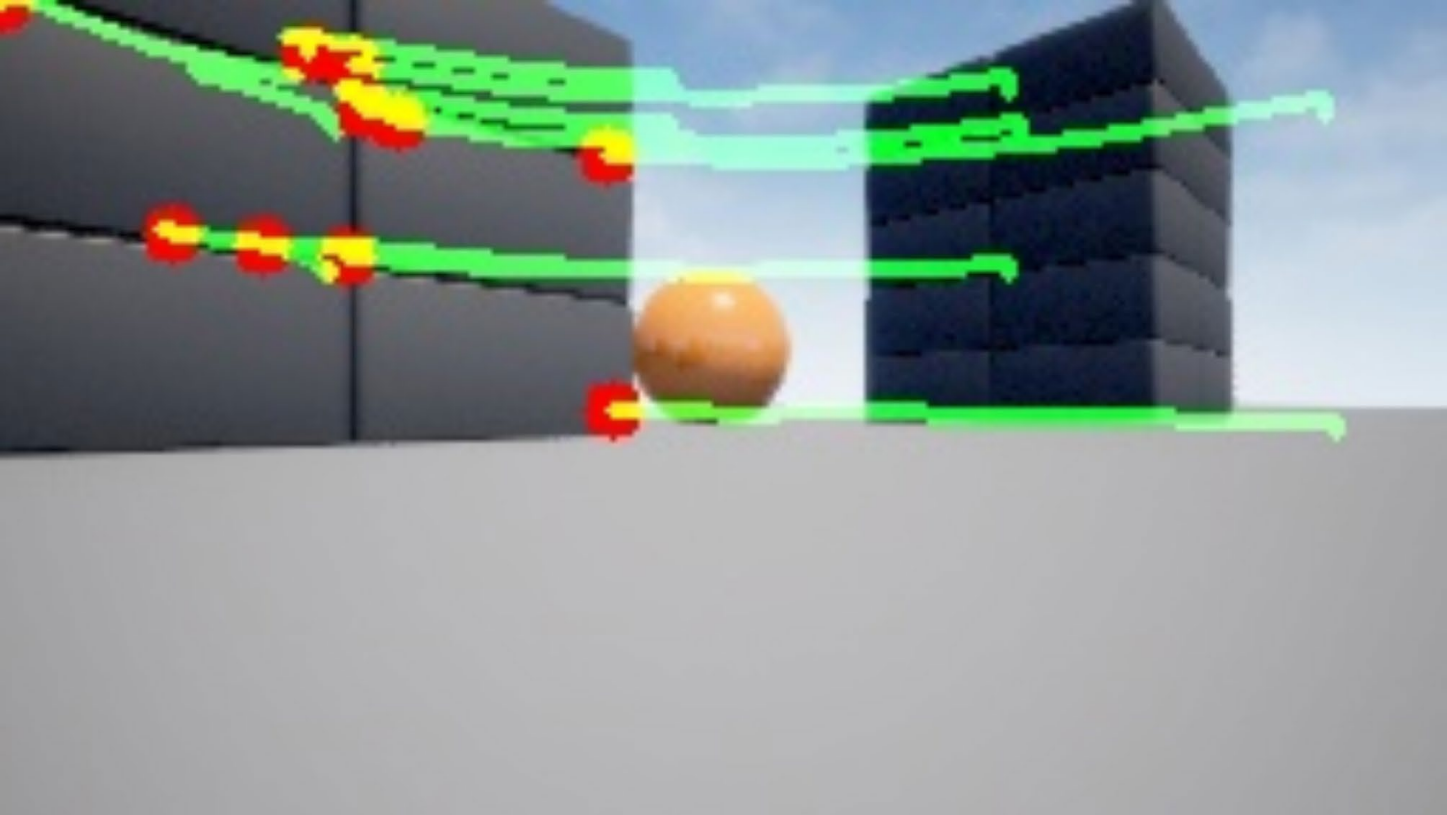
Object Detection and Tracking: Red dots denoting object detection and green Lines indicating the tracking of object’s shift.

Concurrently AirSim’s object detection module scans each frame for objects of interest. Upon detection, the system queries the AI search index to retrieve previous instances of the object. If a match is found and positional parameters align, the object is considered redundant, streamlining the robot’s navigation. However, if the object is not previously recorded, or its position has significantly changed, the image and its sensory data are sent to the ViH model for analysis. To further track changes, we employ Shi-Tomasi corner detection, and the Lucas-Kanade optical flow algorithm as shown in Fig. 5. These updates are then incorporated into the AI search index. Additionally, our system identifies instances where previously detected objects have been removed, ensuring the AI search index accurately reflects the dynamic environment. The working of point cloud object tracking and mapping is discussed in Algorithm 1.

**Algorithm 1:**
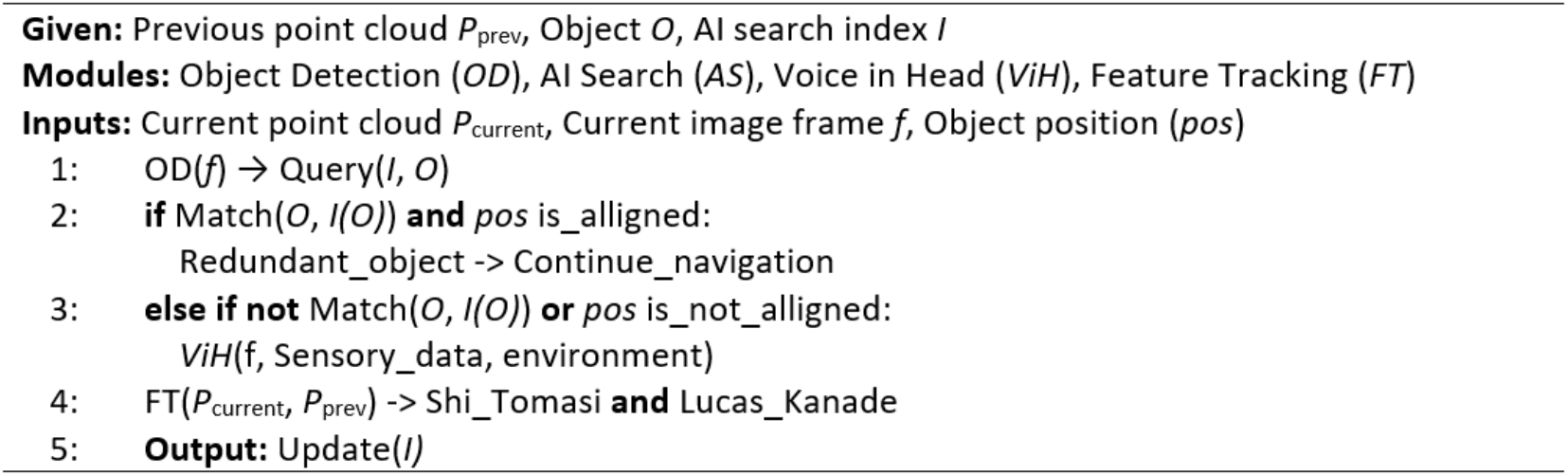
Point cloud object tracking and mapping.

### Voice in head RLHF implementation

We propose the Voice in Head model, a Proximal Policy Optimization (PPO)^34^ eqw based RLHF Framework inspired by the ‘HOLISTIC’ model in neurobiology of language proposed by^35^. This node acts as a control centre, orchestrating the system’s learning process and facilitating training of the agent. It incorporates human feedback to guide the AI’s decision-making for unseen cases. The Actor and Critic LLMs are the driving force behind the RLHF framework. The Actor LLM leverages visual and sensory input from partially observable environment to generate potential actions and inferences in response to the current environmental state and pose queries. The Critic LLM provides constructive feedback by evaluating these actions and observation against ground truth from the environment and answer Actor’s queries. If the Critic is unsure about the Actor’s responses even after continuous feedback, human intervention is triggered to avoid hallucinations. This iterative feedback loop ensures continuous improvement and adaptation of the system’s understanding during the training process. The working of ViH is shown in Algorithm 2.

**Algorithm 2:**
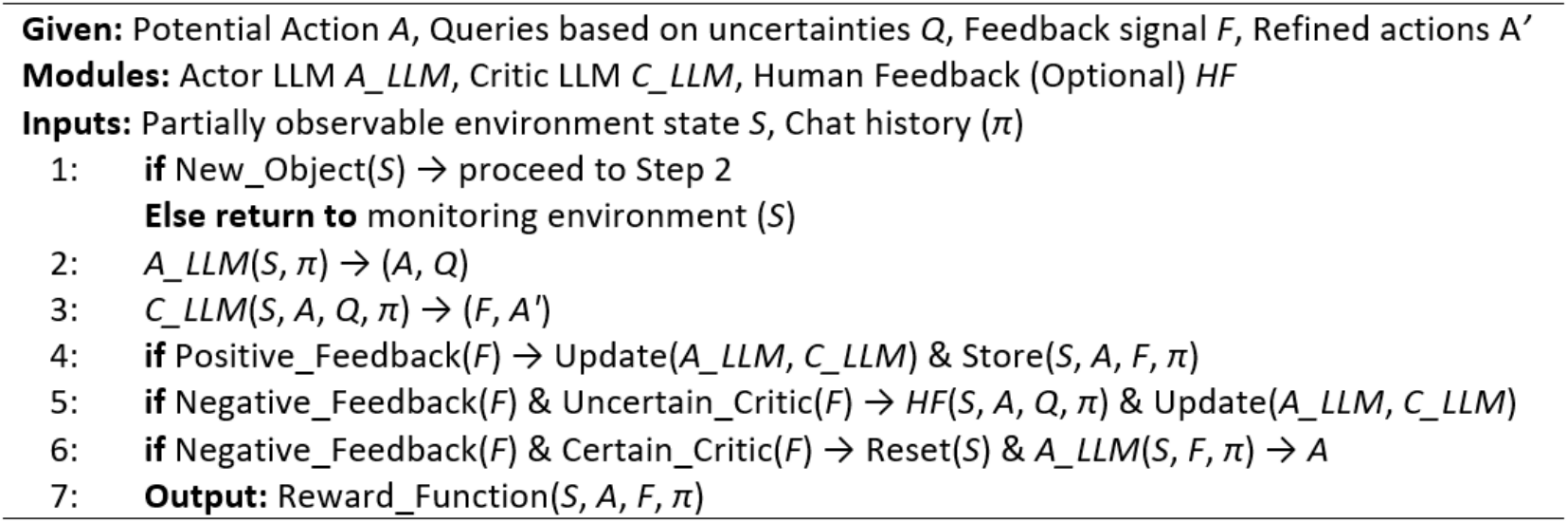
Voice in head.

#### Actor

**Fig. 6:**
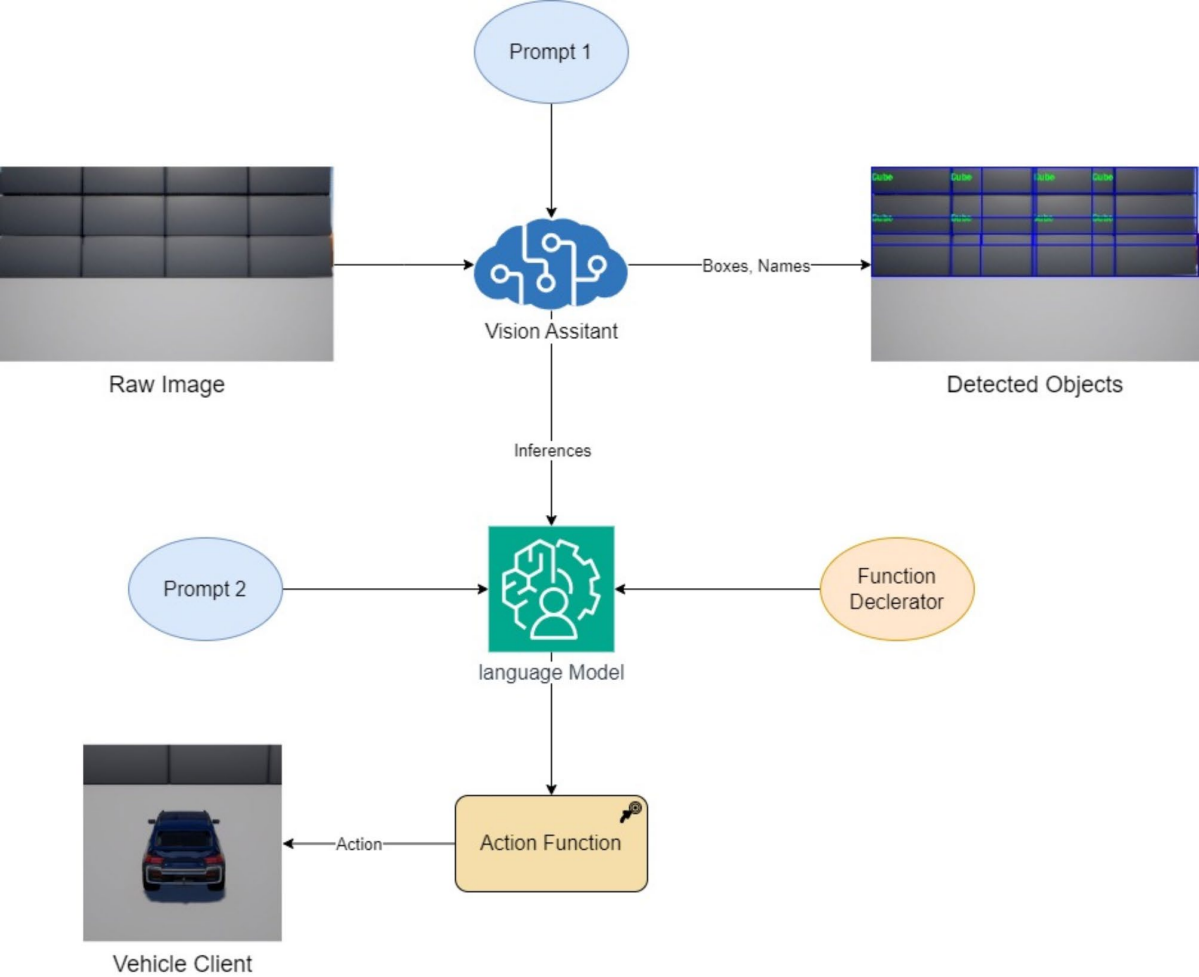
Functional flow diagram of the system proposed system.

The Actor module serves as the system’s perceptual and decision-making core. It leverages a Large Language and Vision Assistant (LLVA) to analyse the environment, generate inferences, and orchestrate actions. We explored both GPT-4, 2023 and Google Gemini Advanced^30^, for their robust natural language understanding and function execution capabilities. These models offer complementary strengths across multiple knowledge domains. After getting the inferences from vision assistant, Actor can perform functions in Eqs. 4, 5 and 6 as shown in Fig. 6 where Prompt 1 contains static instructions about the role of agent and the expected output. Prompt 2 contains dynamic instructions which can change after feedback. It is also equipped with GPS data, car state, lidar inference and positional arguments about objects in the scene using inbuild functions of Vehicle Client in Airsim api.

4 $$Throttle = \left\{ {\begin{array}{*{20}l} {1,} \hfill & {action \ne 0} \hfill \\ {0,} \hfill & {action = 0} \hfill \\ \end{array} } \right.$$

5 $$Brake = \left\{ {\begin{array}{*{20}l} {1,} \hfill & {action = 0} \hfill \\ {0,} \hfill & {otherwise} \hfill \\ \end{array} } \right.$$

6 $$Steering = \left\{ {\begin{array}{*{20}l} {0,} \hfill & {action = 1} \hfill \\ {0.5,} \hfill & {action = 2} \hfill \\ { - 0.5,} \hfill & {action = 3} \hfill \\ {0.25,} \hfill & {action = 4} \hfill \\ { - 0.25,} \hfill & {otherwise} \hfill \\ \end{array} } \right.$$

#### Critic

The Critic LLM plays a pivotal role within the ViH framework, serving as a guiding force in the RL process. It receives the Actor LLM’s proposed action (*A*), the current environmental state (*S*), the Actor’s queries (*Q*), and the chat history (*π*) as mentioned in Algorithm 2. The Critic’s primary responsibility is to evaluate the quality and feasibility of proposed actions, providing the Actor with feedback (*F*) that directly shapes its decision-making and behaviour. This iterative feedback loop fosters continuous learning and adaptation throughout the training process.

The Critic LLM’s evaluation and subsequent feedback are directly incorporated in the reward function:

7 $$\:R\left(t\right)\:=\:\:\alpha\:\:*\:LLM\_Eval(A,\:S,\:Q,\:\pi\:)\:+\:\beta\:\:*\:OD\left(S\right)\:+\:\gamma\:\:*\:R\_Base(S,\:\varDelta\:T)$$

Here, *LLM_Eval*(*A*, *S*, *Q*, *π*) captures the Critic’s output and the Actor’s refinement, while *OD*(*S*) encourages actions leading to object discovery. *R_Base*(*S*, *ΔT*) ensures fundamental elements like speed and distance are considered. The Critic, through its feedback (*F*), influences the reward, guiding the Actor toward optimal decisions.

In situations of high uncertainty, the Critic can initiate human intervention (*HF*). Human experts then provide additional instructions to guide the Critic and Actor LLMs. Critically, the chat history (*π*), which acts as the system’s policy, is directly the incremental updated (*∆π*) based on these interactions:

8 $$\:\varDelta\:\pi\:\:=\:\eta\:\:*\:{\nabla\:}_{\pi\:}{\varSigma\:}_{t}R\left(t\right)+\:log\left(\pi\:\right)$$

Where *η* which usually is the learning rate but here, it’s the compression rate to keep token usage under limit. Compression is done by summarization of previous content based on their cumulative rewards *R(t)*, for time *t*. *log(π)* represents the latest entry to the chat which is also quantized.

Through these updates, the Critic learns alongside the Actor, leading to increasingly robust decision-making. Let’s assume a discount factor *δ* which controls the importance of future rewards, the cumulative reward at the end of an episode is given by:

9 $$\:R\_Final\:=\:\varSigma\:\_t\:\:\delta\:^(t-1)\:\:*\:R\left(t\right)$$

### Object-Oriented semantics

Object-oriented semantics provides a powerful framework for representing and managing the knowledge acquired by autonomous systems during navigation. In this approach, entities within the environment (such as vehicles, landmarks, or obstacles) are modelled as objects with associated properties and relationships. This structured representation mirrors how humans intuitively perceive and interact with the world, establishing a common ground between machine and human understanding. Crucially, object-oriented semantics facilitates several key benefits for robotic perception and decision-making. By defining objects and their interrelationships, object-oriented semantics allow an autonomous system to reason about the broader context in which it operates, rather than just reacting to isolated data points. The structured nature of this approach promotes efficient storage, retrieval, and updating of information within a knowledge base, enabling the system to learn from past experiences and adapt its behaviour. Furthermore, the ability to reason about objects with defined properties can significantly reduce time complexity in navigation tasks, as the system can make more informed decisions based on its structured understanding of the environment.

#### Azure AI search

We leverage Azure AI Search as a centralized knowledge repository to manage the object-oriented semantic data acquired throughout navigation. Within this repository, we store diverse data elements crucial for decision-making, including raw visual input from the vehicle’s cameras, calculated rewards reflecting episodic performance, positional arguments related to both the vehicle and detected objects, and supplementary sensory data. Importantly, we structure the information within AI Search around episodes, where each episode serves as a complete navigation cycle. This organization enables the system to retrieve and reason about contextual information during learning and task execution.

#### Language transformation

To ensure the data stored within Azure AI Search is enriched with contextual understanding, we employ a dedicated Language Transformation LLM. This LLM’s primary responsibility is to analyse the entirety of the episodic conversations, which include the interactions between the Actor and Critic LLMs, as well as any human intervention instances. The LLM extracts salient semantic elements, encompassing object descriptions, actions performed, and overall episode goals. Subsequently, this information is transformed into structured JSON properties. These properties are then integrated alongside images, rewards, and sensory data within Azure AI Search, adding a layer of contextual depth to the stored information.

#### AI search query with LLM frontend

For user-friendly interaction with the accumulated semantic data, we implement a synergistic combination of Azure AI Search’s query mapping capabilities and a front-end LLM. AI Search excels at translating natural language user queries into structured search requests. The LLM frontend functions as an intelligent user interface, equipped with Natural Language Processing (NLP) capabilities to decipher the intent behind user queries. It collaborates with the query mapper by mapping the user’s query to pre-defined functions within the AI Search API. These targeted searches within the knowledge base facilitate efficient retrieval of the most relevant information in response to the user’s natural language query.

## Experimental results

**Fig. 7:**
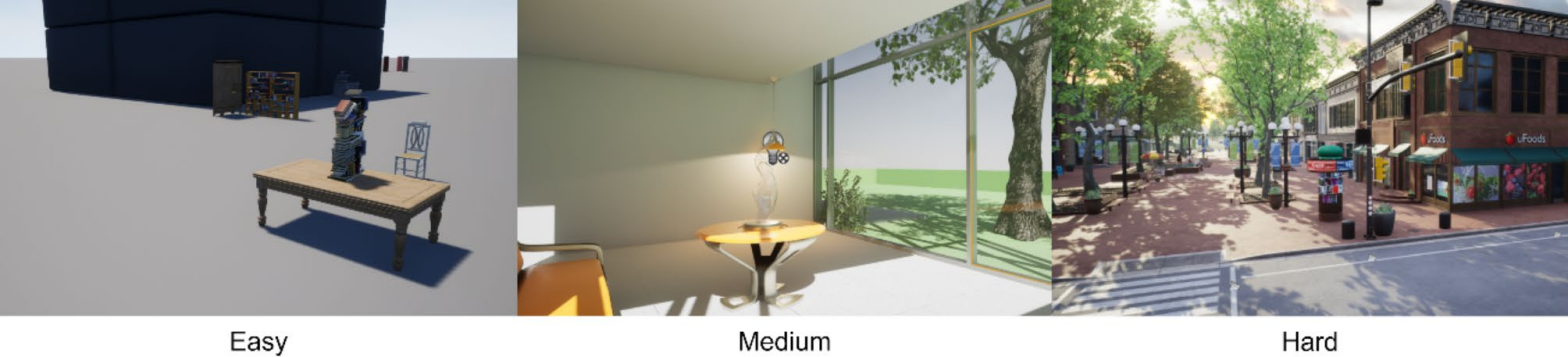
Unreal engine simulation environments used during experimenting^10,11^.

To comprehensively assess the capabilities of our proposed system, we designed a series of controlled experiments within simulated environment. This simulation setting offers the advantages of precise environmental control, the ability to test a vast array of scenarios, and safe, risk-free experimentation that is crucial for autonomous systems. We designed a tiered experimental setup to progressively test the system in environments of increasing complexity. As illustrated in Fig. 7, we employed three distinct environments: the “Blocks” environment enhanced with strategic asset additions for simpler complexity, Unreal Engine’s sample modern house project replicating a cluttered indoor setting for moderate complexity, and finally, the “Downtown West” environment simulating a dynamic outdoor urban landscape.

### Object tracking

Our evaluation for this section focuses on the effectiveness of triggering the ViH model pipeline during navigation and mapping. We assess this by examining “Hits” and “Misses” in triggering the pipeline for each individual instance. A “Hit” signifies a successful triggering when the system correctly identified the need to activate the ViH model. Conversely, a “Miss” indicates that the system failed to trigger the ViH model when it was necessary. It’s important to note that we are primarily concerned with the overall triggering success rate, rather than dissecting the specific causes of misses (whether due to object tracking or semantic search or object detection failures). This is because isolating these individual factors falls outside the scope of our current research. The number of Hits account to the number of episodes an agent will go through to analyse the whole map.

Table 1 summarizes the number of trigger instances across the three environments:

**Table 1:** Triggering summary during mapping.

Environment	Total trigger instances	Hits	Misses
Easy	179	165	14
Medium	524	471	53
Hard	1067	883	184

A hit accuracy of 92.178%, 89.885% and 82.755 for easy, medium, and hard levels respectively. Here total trigger instances are determined by the number of unique objects spread across the environment. These values are determined using ground truth from the environment.

### Navigation

To comprehensively assess the navigation capabilities of our proposed model, we conducted a comparative evaluation against AirSim’s built-in Deep Q-Network (DQN) script and the Proximal Policy Optimization (PPO) algorithm. Both baseline models were configured to operate with the same trigger conditions as our ViH model, powered by GPT-4 and Gemini Pro (tested separately). We consider each trigger instance equivalent to a RL episode. To account for variations in internet connectivity and server response times, we focused on recording the internal processing time of each model for an episode, excluding the time spent on API requests. It’s worth noting that ViH-GPT utilizes GPT-V for initial vision analysis before sending results to GPT-4, while ViH-Gemini employs a single Gemini Pro model for combined vision and semantic understanding.

**Fig. 8:**
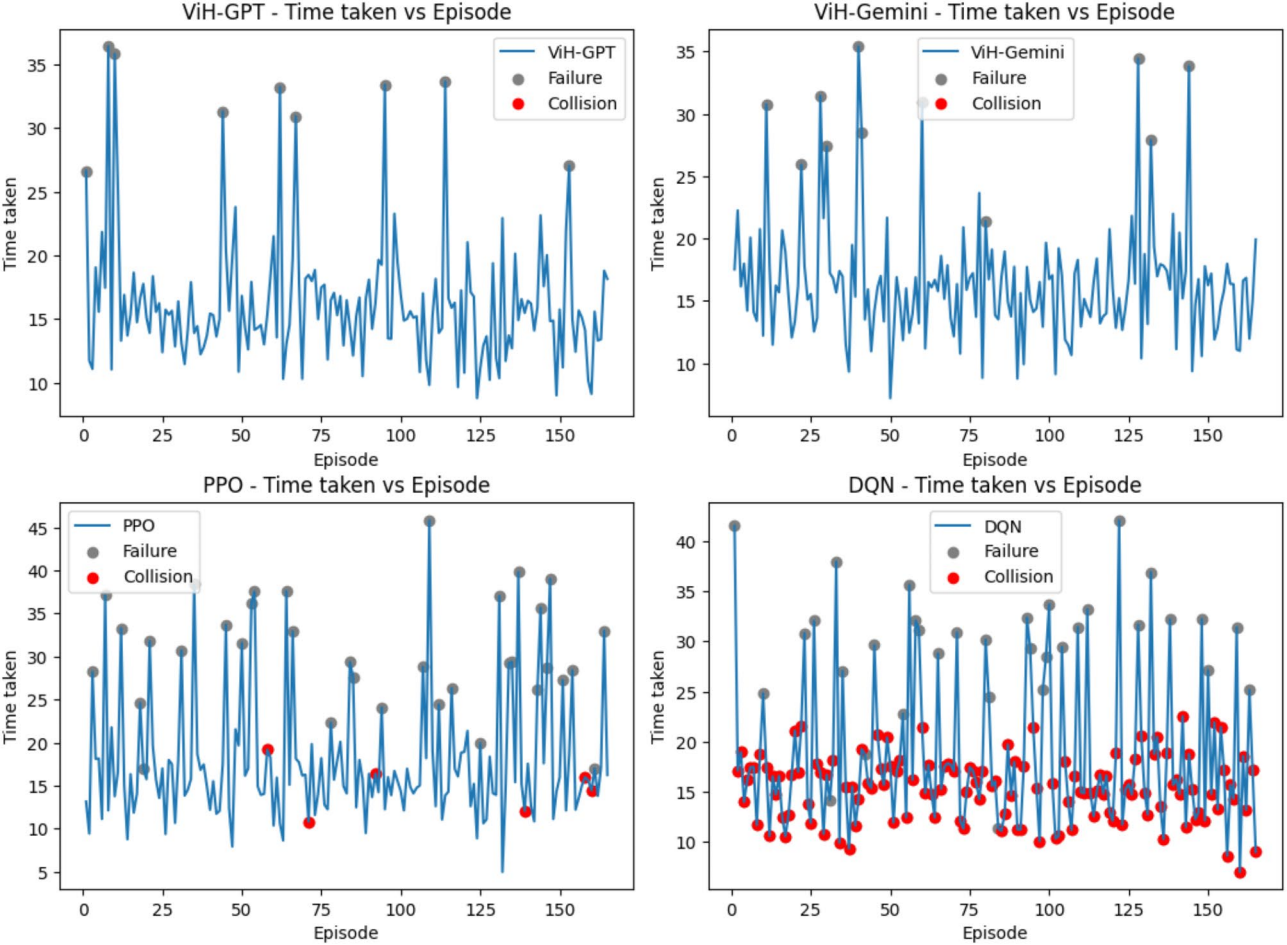
Time taken per episode with failures and collisions that took place for “Easy” level.

**Fig. 9:**
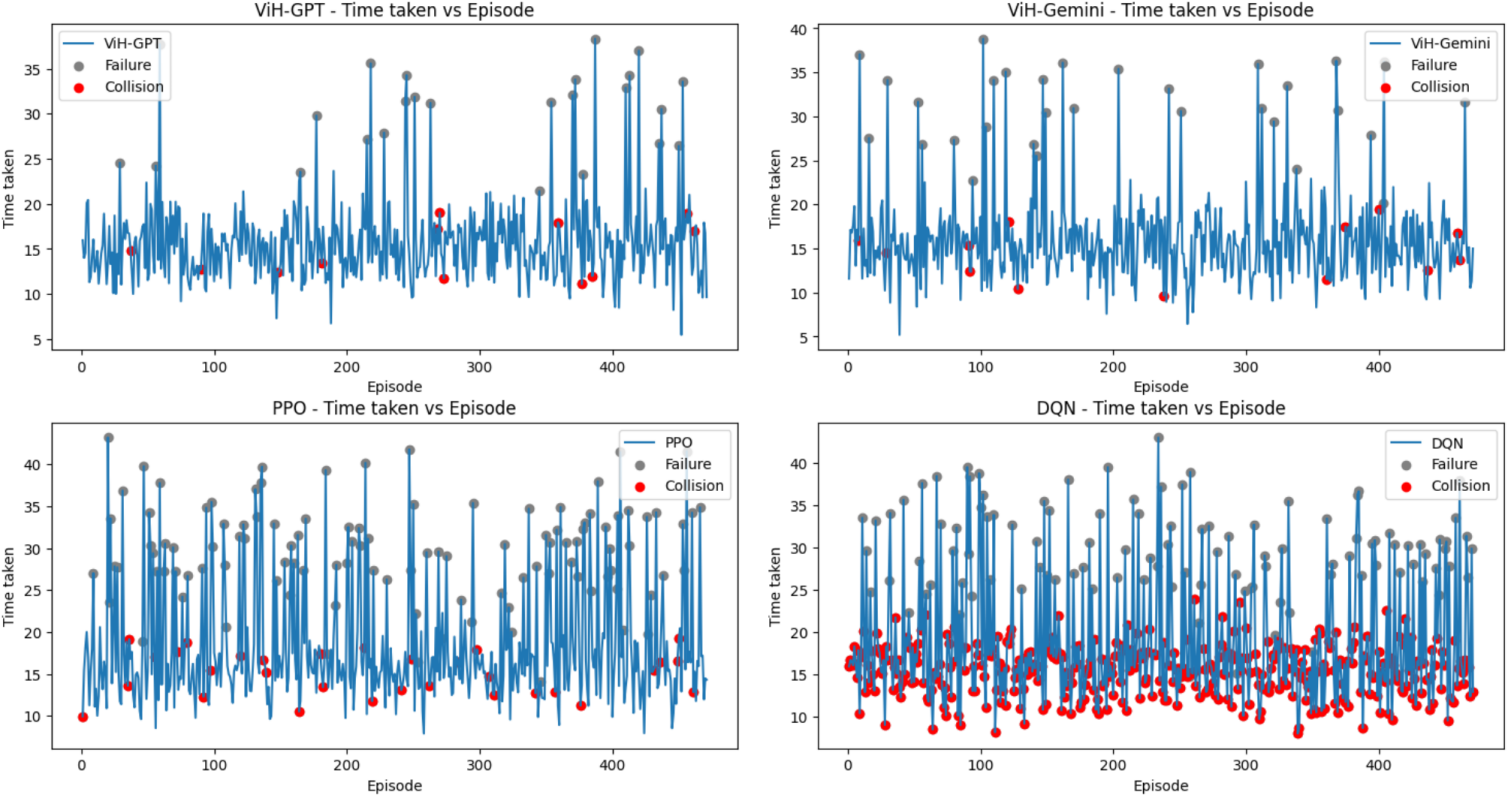
Time taken per episode with failures and collisions that took place for “Medium” level.

**Fig. 10:**
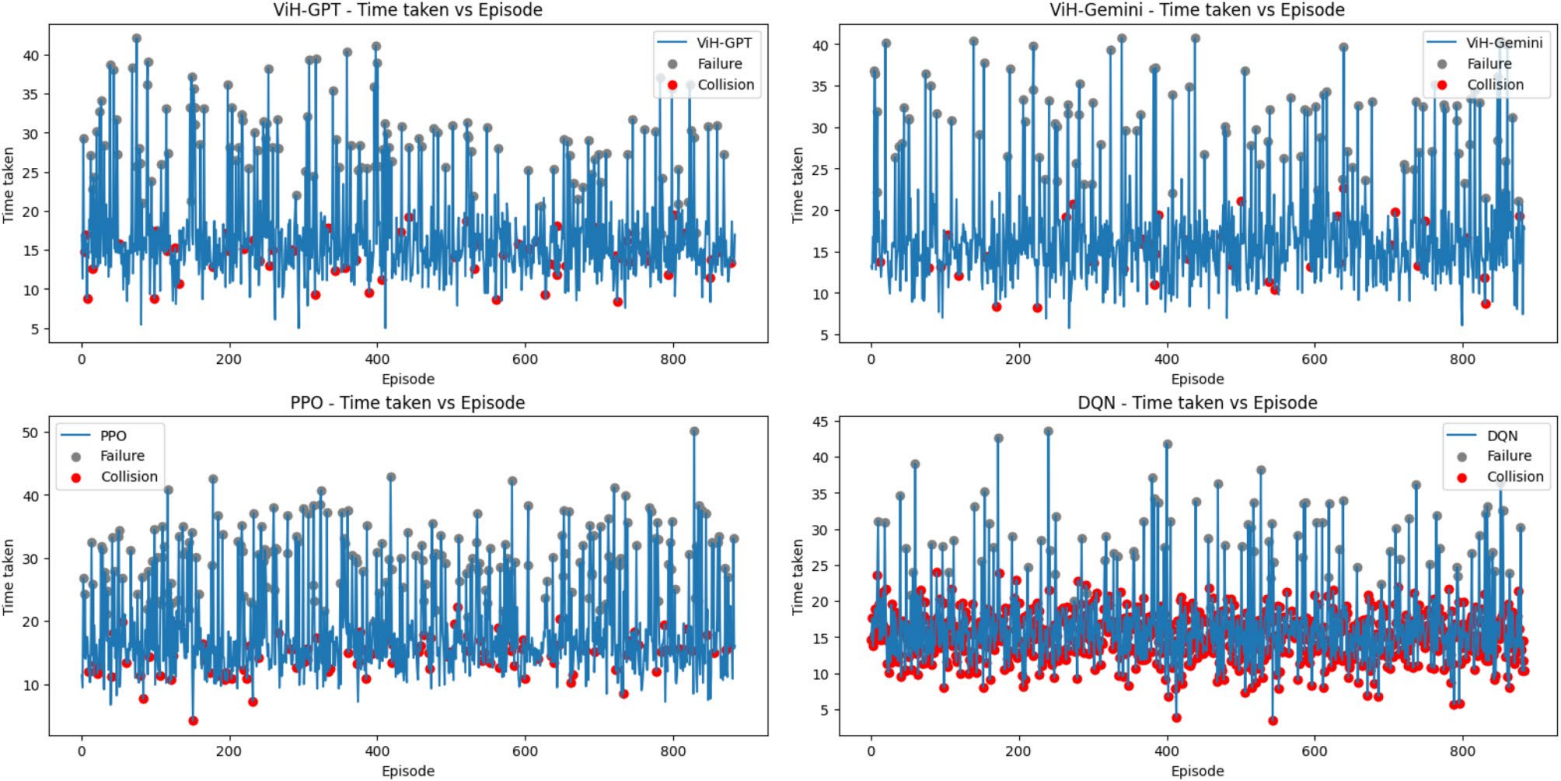
Time taken per episode with failures and collisions that took place for “Hard” level.

**Fig. 11:**
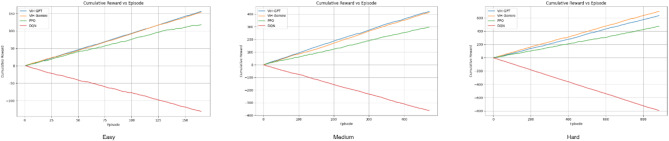
Cumulative rewards per episode for all the models in all 3 environments.

For all three difficulty environments, we tracked internal processing time per episode along with episode outcomes indicating success or failure (Figs. 8, 9 and 10). Within these figures, grey dots represent failed episodes, while red dots signify episodes terminated due to collisions. Additionally, we monitored timesteps throughout the training process. Each timestep corresponds to a single action taken within an episode, providing a granular measure of performance. Finally, rounded off cumulative rewards (calculated using Eq. 9 with a discount factor, *δ* = 0.2) acquired over training for all models in each environment are presented in Fig. 11. Here, the episodes having successful execution were observed to receive a reward around + 1 while the rest received a reward around 0. Episodes undergoing collision received a reward around − 1.

Table 2 presents a detailed comparison between the four navigation models across all three environments. The following four key metrics form the basis of our evaluation:

- *Success Rate (%)*: The percentage of episodes where the navigation agent successfully reached the designated goal within a predefined time limit.
- *Collision Rate (%)*: The frequency of collisions with obstacles during navigation, expressed as a percentage of the total episodes for that level.
- *Total Timesteps*: The cumulative number of atomic actions taken by an RL agent during navigation. This granular measurement reflects the overall execution time of all episodes for a given environment.
- *Final Reward*: The summation of all cumulative rewards, offering insights into the ability of a model to learn and optimize decisions over time.

**Table 2:** Comparison of ViH model with traditional models for navigation through the environment.

Level	Metrics	ViH-GPT	ViH-Gemini	PPO	DQN
Easy	Success Rate (%)	**94.54**	93.33	75.15	0.00
	Collision Rate (%)	**0.00**	**0.00**	3.63	79.39
	Total Timesteps	**2**,**689**	2,709	3,000	3,014
	Final Reward	**156**	154	118	− 131
Medium	Success Rate (%)	**92.14**	90.66	70.06	0.00
	Collision Rate (%)	**2.55**	2.76	6.58	76.86
	Total Timesteps (seconds)	**7**,**428**	7,546	8,683	8776
	Final Reward	**422**	414	299	− 362
Hard	Success Rate (%)	78.93	**83.57**	65.91	0.00
	Collision Rate (%)	7.13	**4.64**	12.23	89.92
	Total Timesteps (seconds)	14,836	14,754	16,147	**14**,**517**
	Final Reward	634	**697**	474	-794

### Semantic query comprehension and management

This section explores how our system interprets and manages natural language queries, both internally generated and those provided by users. We analyse the average number of tokens employed by the LLMs, particularly GPT-4 and Gemini Pro, during training episodes and when responding to user queries. Critically, we examine how effectively the LLMs’ chat histories are summarized when reaching their respective token limits. This summarization technique is essential for maintaining a manageable history size within the ViH system. Table 3 presents a comparative analysis, detailing the average token usage and the associated compression percentages achieved for GPT-4 and Gemini Pro for different query types. Since GPT-V’s chat history is refreshed with every usage, eliminating the need for compression.

**Table 3:** Average tokens used by LLMs per training episode and user query vs. compression percentage after reaching token limit.

LLM	Max Tokens	Average Token Usage		Compression (%)
		Training Episode	User Query	
GPT – 4	32,768	4,532	1,586	86.2
GPT – V	128,000	87,294	56,243	–
Gemini Pro	30,720	2,976	1,872	90.04

Following extensive training, we evaluated the performance of both ViH-GPT and ViH-Gemini models in accurately interpreting and responding to diverse user queries. This evaluation focused solely on the overall system’s ability to handle queries, independent of the specific environments the models were trained in. Table 4 summarizes the percentage of correct responses for retrieval and actionable queries, further categorized by complexity as follows:

- Simple Retrieval Queries (Total Queries: 80):
  - “Where is the nearest ufoods?”
  - “Is there a tree near you?”
  - “What is the colour of table in living room?”
  - Example – Where is the nearest ufoods?
    - *Function Call -* *triggerAzureAISearch*(“nearest ufoods”).
    - *Response (ViH-GPT) -* “The nearest ufoods is at coordinates (28.7041, 77.1025), approximately 2.5 kilometers north. Proceed along Main Street and turn left at the second intersection.”
    - *Response (ViH-Gemini) -* “You can find the nearest ufoods at coordinates (28.7041, 77.1025), 2.5 kilometers away. Head north for about 2 kilometers, then take a left turn at Baker’s Lane.”
- Complex Retrieval Queries (Total Queries: 40)
  - “Find me a quiet place to sit.”
  - “Recommend a restaurant serving Italian cuisine with good vegetarian options nearby.”
  - “Suggest me something to wear based on the weather?”
  - Example – Recommend a restaurant serving Italian cuisine with good veg options nearby.
    - *Function Call -* *triggerAzureAISearch*(“restaurant serving Italian cuisine with good vegetarian options nearby”).
    - *Response (ViH-GPT) -* “I recommend ‘La Bella Italia,’ located at coordinates (148.362, 8.023), known for its excellent vegetarian pasta and pizza. It is 1.2 kilometers west, right after the city park.”
    - *Response (ViH-Gemini) -* “Try ‘La Bella Italia,’ at coordinates (148.362, 8.023), which has great Italian vegetarian dishes. It’s 1.2 kilometers west of your location, just past the central library.”
- Image-Based Retrieval Queries (Total Queries: 20):
  - User uploads an image of a specific object. *“Where is this object?”*
  - User uploads an image of a room. *“Was there any book in this room?”*
  - User uploads an image of an award. *“Was this award on the table?”*
  - Example – Where is this object?
    - *Function Call -* *triggerImageSearch*(img.jpg, query ).
    - *Response (ViH-GPT) -* “This object is on the second shelf of the bookcase in the living room at coordinates (8.232, 65.420). Look next to the blue photo frame.”
    - *Response (ViH-Gemini) -* “The object is on the second shelf of the bookcase in the living room at coordinates (8.232, 65.420). It’s next to the green vase.”
- Simple Actionable Queries (Total Queries: 40):
  - “Go to living room.”
  - “Go near the tree.”
  - Example – Go to living room.
    - *Function Call -* *triggerAzureAISearch*(“living room location”).
    - *Action -* *gotoCoordinates*(10.021, 60.204).
- Complex Actionable Queries (Total Queries: 20):
  - “Goto any animal statue but take a longer route.”
  - “I want to wash my hand, lead me to it.”
  - Example – I want to wash my hand, lead me to it.
    - *Function Call -* *triggerAzureAISearch*(“nearest washroom location”).
    - *Action -* *gotoCoordinates*(15.852, 69.481).
- Image-Based Actionable Queries (Total Queries: 10):
  - User uploads an image of a cluttered desk. *“Goto this workspace.”*
  - User uploads an image of a light fixture. *“Go to the switch that will turn this light off.”*
  - Example – *Go to the switch that will turn this light off*.
    - *Function Call -* *triggerImageSearch*(img.jpg, query).
    - *Action -* *gotoCoordinates*(7.119, 64.696).

**Table 4:** Response analysis for all queries.

Model	Retrieval Queries (%)			Actionable Queries (%)		
	Simple	Complex	Image	Simple	Complex	Image
ViH-GPT	95.00	85.00	85.00	**97.50**	80.00	90.00
ViH-Gemini	**95.00**	82.50	80.00	**95.00**	70.00	80.00

For retrieval queries, responses focused on the use of semantic search (LLM function calls combined with Azure AI Search). Actionable queries required the system to both retrieve information and perform actions to fulfill the request. To assess the accuracy of the models’ responses, results for ViH-GPT and ViH-Gemini were compared against a manually verified ground truth.

### Human feedback component

**Fig. 12:**
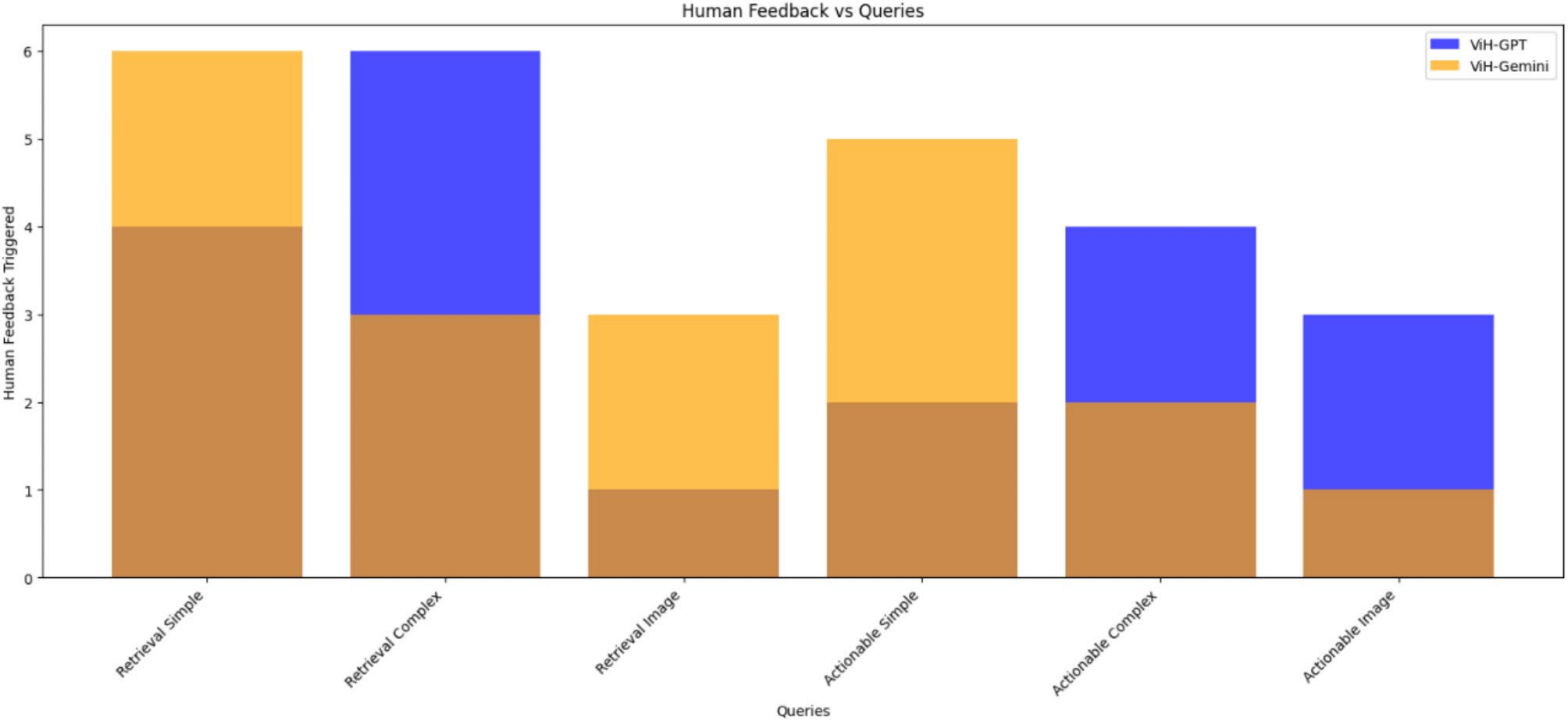
Number of times Human feedback was triggered for different types of queries by ViH-GPT.

This component plays a critical role in refining the model’s responses. Human interventions are triggered in instances where the system’s confidence in its response is low or when the provided response is identified as potentially incorrect. The nature and frequency of these interventions are illustrated in Fig. 12, which compares the instances of human feedback required for different query types by ViH-GPT and ViH-Gemini.

In our analysis, we found that ViH-GPT required more human feedback compared to ViH-Gemini. Specifically, ViH-GPT called for human feedback approximately 33% more frequently across various query types. This difference highlights ViH-GPT’s greater reliance on human intervention to ensure response accuracy, particularly in complex and image-based queries. For instance, ViH-GPT needed more feedback for complex retrieval and actionable queries, indicating challenges in these areas.

## Discussion

In this section, we delve deeper into the experimental results presented earlier and explore the implications for our ViH system. We analyse the performance trends between ViH-GPT and ViH-Gemini, focusing on their efficiency in query handling, responsiveness to user requests, and adaptability when facing challenging queries. Additionally, we examine the limitations revealed during the evaluation, providing insights for future refinements and potential avenues for enhancing the system’s autonomous capabilities.

### Object tracking

The tiered approach adopted for evaluating object tracking proved valuable in assessing the system’s ability to learn and adapt its navigation strategies across environments with varying difficulty levels. This evaluation provided comprehensive performance benchmarks, highlighting the system’s strengths and areas for potential improvement under real-world complexities. Notably, the modular design of the approach facilitates replication for future research endeavors.

The results for triggering the ViH model pipeline during navigation and mapping are promising. The achieved hit accuracy indicates a high success rate in correctly identifying situations that necessitate ViH model activation (ranging from 92.178% in easy environments to 82.755% in hard environments). It’s important to remember that these “misses” where the system failed to trigger the pipeline do not encompass the specific reasons for the failures, such as limitations in object tracking, semantic search, or object detection. A deeper dive into these potential causes might be a consideration for future investigations.

### Navigation

The comparative evaluation reveals prominent differences in the navigation capabilities of the tested RL models: ViH-GPT, ViH-Gemini, Simple PPO, and AirSim DQN. Our proposed ViH models consistently outperform both baseline models (PPO and DQN) across all difficulty levels in terms of success rate, collision rate, and cumulative reward. This highlights the advantages of incorporating LLMs for both the Actor and Critic roles within the RL framework, as they appear to offer significant improvements in decision-making and navigation compared to more traditional RL models. Furthermore, the ViH models demonstrate a remarkable ability to navigate complex environments while minimizing collision rates. This suggests a superior understanding of spatial relationships and the ability to avoid obstacles effectively, potentially enabled by the rich semantic information the LLMs incorporate into their decision-making processes.

It was observed that DQN failed all the tests with huge collision rates given the model was equipped with no semantic understanding and decision-making working on randomized concepts which weren’t suitable for the given episode frames. The PPO implementation had more information about the sensory data to perform more informed decisions The observed dominance of ViH-GPT in easy and medium environments suggests that separating vision processing (with GPT-V) and language understanding (with GPT-4) offers advantages in less complex settings. This modularity might enable faster decision-making and a more efficient use of language modelling capabilities. Whereas ViH-Gemini outperformed the other models in the hard environment. This indicates that combining vision and language processing within a single LLM can be particularly beneficial when navigating highly intricate surroundings, where understanding spatial context and visual cues is important. When analysing these metrics, it’s crucial to consider them as an interconnected system. A high success rate coupled with a low collision rate indicates that the model navigates effectively. Total timesteps should be analysed in the context of reward – a lower number of timesteps combined with high final reward implies efficiency in the model’s navigation strategy.

It was also observed that episodes terminated faster when a collision occurred and took more time when a failure occurred without any collision due to stalling in Figs. 8, 9 and 10. Further it can be observed in Fig. 11 that due to the approximation in result values to keep it simpler for LLM’s understanding, the cumulative reward vs. episode graph shows a close to linear representation for all the models, where the larger is the positive slope the more efficient is the model. DQN showed a negative slope in all the scenarios.

### Semantic query comprehension and management

Our analysis of LLM token usage from Table 3 reveals significant differences between GPT-4 and Gemini Pro. These differences highlight how each LLM processes and manages information. GPT-4, had a larger token allowance and utilized more tokens during training episodes on an average which could be due to it getting inferences from GPT-V separately which in case Gemini Pro is integrated within one mode as this opposite in the case of average token usage to process user queries. Its higher compression percentage suggests an ability to generate concise responses. Gemini Pro offered a higher compression ratio of 90.04% which means that whenever it reached its token limit it summarized the chat history to 9.96% of its original size. GPT-V, on the other hand, consistently uses a substantially greater proportion of its available tokens. While these differences in token usage patterns might lead to variations in response generation style (e.g., verbosity, conciseness), further investigation is needed to correlate these patterns with specific qualitative aspects of the models’ responses.

The ViH system, particularly in the ViH-GPT configuration, demonstrates high accuracy in handling both retrieval and actionable queries (Table 4). Across query complexity levels (simple, complex, and image-based), the system consistently maintains a reliable level of performance. This robustness showcases the power of LLMs to understand natural language instructions and effectively combine semantic search with the ability to initiate actions within the environment. It’s worth noting that, in certain cases, image-based queries might have achieved greater accuracy due to the potential effectiveness of Azure AI Search in handling visual search tasks.

The ViH system demonstrates impressive capabilities in understanding and responding to various user queries. However, the analysis of instances where human intervention was needed reveals a key focus area for improvement. By understanding the specific circumstances triggering human feedback, we can pinpoint potential shortcomings in the LLMs’ semantic understanding or reasoning processes. Importantly, our approach, incorporating LLMs alongside a human feedback mechanism, has demonstrably reduced the frequency and impact of LLM hallucinations.

## Conclusion

The development of the ViH system offers compelling insights into the intersection of robotics, LLMs, and the pursuit of artificial general intelligence (AGI). This research highlights the transformative potential of LLMs when strategically integrated into autonomous robotic systems. By empowering a robot to understand natural language instructions, leverage semantic search, and initiate contextually relevant actions, we take a significant step towards systems that operate more flexibly and intuitively within real-world environments. Our exploration underscores the intricate relationship between language understanding, semantic representation, and embodied action. The ViH framework’s success in grounding language commands within a physical environment suggests that semantic grounding, rather than solely relying on massive language datasets, could play a pivotal role in developing more robust AGI systems. Furthermore, the demonstrated advantages of modularity, as seen with the separation of visual processing and language modeling in ViH-GPT, call for a careful consideration of architectural design choices in intelligent systems.

Crucially, our experimental findings illustrate the continued value of human feedback in refining and guiding LLM-based systems. While LLMs exhibit remarkable capabilities, their tendency towards hallucination necessitates careful oversight. The integration of RLHF within the ViH system offers a promising avenue for mitigating these challenges. This approach highlights the potential for hybrid systems, those that leverage both the strengths of powerful LLMs and the nuanced understanding of human input, in the pursuit of AGI. As we forge ahead, it is vital to acknowledge the limitations of purely data-driven approaches. While LLMs excel at pattern recognition and language generation, they often struggle with complex reasoning, common-sense understanding, and generalization to novel scenarios. This work suggests that future advancements in AGI might necessitate a deeper exploration of knowledge representation, symbolic reasoning, and the integration of diverse cognitive abilities. The ViH system represents a significant contribution to the fields of robotics and intelligent system design. This research paves the way for further exploration into the role of LLMs, semantic grounding, modular architectures, and the nuanced interplay between human guidance and AI capabilities as we strive for increasingly intelligent and adaptable robotic systems. This work opens up several promising avenues for future research which include conducting in-depth profiling of GPT-4 and Gemini Pro, examining factors such as response length, accuracy, and processing efficiency, would provide a deeper understanding of their respective strengths and weaknesses for different query types. Also, the work can focus more on targeted data augmentation by expanding the training datasets with complex queries and scenarios where hallucinations may occur could improve the models’ robustness and reduce the need for human intervention. Another futuristic scope will be towards developing intelligent techniques for identifying when human feedback is truly necessary, as well as exploring alternative reward functions within the RLHF framework, could further optimize the human-AI collaboration within the ViH system.

## Data Availability

No human or human data was used in this work. The data used in this work is available in the following links: https://github.com/microsoft/AirSim/tree/main/Unreal/Environments/Blockshttps://www.unrealengine.com/marketplace/en-US/product/realistic-renderinghttps://www.unrealengine.com/marketplace/en-US/product/6bb93c7515e148a1a0a0ec263db67d5b.
